# Pattern Recognition Receptors and Autophagy

**DOI:** 10.3389/fimmu.2014.00300

**Published:** 2014-06-25

**Authors:** Ji Eun Oh, Heung Kyu Lee

**Affiliations:** ^1^Laboratory of Host Defenses, Graduate School of Medical Science and Engineering, Korea Advanced Institute of Science and Technology, Daejeon, South Korea

**Keywords:** autophagy, toll-like receptors, RIG-I-like receptors, NOD-like receptors, inflammasomes, cytosolic DNA sensors

## Abstract

The immune system senses exogenous threats or endogenous stress through specialized machinery known as pattern recognition receptors (PRRs). These receptors recognize conserved molecular structures and initiate downstream signaling pathways to control immune responses. Although various immunologic pathways mediated by PRRs have been described, recent studies have demonstrated a link between PRRs and autophagy. Autophagy is a specialized biological process involved in maintaining homeostasis through the degradation of long-lived cellular proteins and organelles. In addition to this fundamental function, autophagy plays important roles in various immunologic processes. In this review, we focus on the reciprocal influences of PRRs and autophagy in modulating innate immune responses.

## Introduction

Innate immune signaling pathways are initiated when microorganism-specific pathogen-associated molecular pattern (PAMP) molecules are recognized by host pattern recognition receptors (PRRs) (1). PRRs can be classified based on their site of localization (e.g., plasma membrane, endosomal vesicles, and cytoplasm) or by molecular structural similarities. PRRs classified by structural similarity include toll-like receptors (TLRs), nucleotide oligomerization domain (NOD)-like receptors (NLRs), C-type lectin receptors (CLRs), and RIG-I-like receptors (RLRs).

The TLRs, which reside both within the cell surface membrane (TLR 1, 2, 4, 5, and 6) and in endosomal compartments (TLR 3, 7, 8, and 9), are the most well-characterized PRRs. After recognition of PAMPs, TLRs initiate downstream signaling pathways via myeloid differentiation primary response gene 88 (MyD88) or Toll/interleukin (IL)-1 receptor (TIR) domain-containing adapter-inducing interferon (IFN)-β (TRIF), ultimately activating the transcription factors nuclear factor (NF)-κB and activator protein-1 (AP-1) or IFN regulatory factor 3 (IRF3). Activation of NF-κB and AP-1 results in the production of proinflammatory cytokines, and activation of IRF3 results in the production of type I IFNs (2). NLRs are cytoplasmic members of the PRR family, and more than 20 NLRs have been identified in mammals. NOD1 and NOD2 – the first NLRs identified in mammals – recognize cytoplasmic bacterial cell wall components, eventually activating NF-κB to induce the production of proinflammatory cytokines. In addition, NLRs act as sensory proteins in inflammasomes (which serve as platforms for protein complexes involved in innate immunity) and activate inflammasome-associated caspase-1 for pro-IL-1β and pro-IL-18 processing. RLRs and other cytosolic sensors primarily recognize microbial nucleic acids in the cytosol. RLRs composed of retinoic acid-inducible gene I (RIG-I) and melanoma differentiation-associated gene 5 (MDA-5) are caspase-recruiting domain (CARD)-containing RNA helicases that recognize double-stranded RNA and signal through IFN-β promoter stimulator-1 [IPS-1; also known as mitochondrial antiviral signaling (MAVS), virus-induced signaling adaptor (VISA), or Cardif] to subsequently activate IRF3 and NF-κB (3).

Autophagy is a highly conserved homeostatic process in eukaryotic cells that degrades long-lived cellular proteins and organelles. There are at least three types of autophagy: microautophagy, chaperone-mediated autophagy, and macroautophagy (4). During microautophagy, continuous degradation of cytosolic constituents close to the lysosomes occurs through budding of the lysosomal membrane. In chaperone-mediated autophagy, proteins containing a “KFERQ” motif are transported into the lysosomal lumen via Lamp2a for subsequent degradation. During this process, cytosolic chaperones such as HSC70 recognize the KFERQ motif and facilitate importation of substrates into the lysosomes (5). Macroautophagy, which is the primary route of degradation, involves the formation of a double-membrane vesicle known as an autophagosome. During this process, long-lived cellular components are first surrounded by an elongated cup-shaped membrane that forms the autophagosome, which then matures and fuses with lysosomes for degradation of the internalized materials (6). Recent research has suggested that autophagy is a selective process, in which specific adaptors such as p62 target ubiquitinated substrates for selective degradation (7).

The molecular processes involved in autophagy consist of three distinct stages. Initiation of isolation membrane formation requires complex interaction between autophagy-related gene *(Atg) 6* (also known as beclin-1) and type III [phosphatidylinositol 3-kinase (PI3K). Elongation of the isolation membrane and termination of autophagosome formation are regulated by at least two ubiquitin-like molecules: microtubule-associated protein 1 light-chain 3 (LC3; mammalian homolog of yeast Atg8) and Atg12 (8, 9). Atg12 is conjugated to Atg5 through the sequential actions of the E1- and E2-like enzymes Atg7 and Atg10. Association of Atg12–Atg5 conjugates with Atg16 in turn facilitates elongation of the isolation membrane and catalyzes LC3 conjugation. The C-terminal amino acids of LC3 are cleaved by Atg4 and then transferred to phosphatidylethanolamine (PE) in the newly formed isolation membrane by the E1- and E2-like enzymes Atg7 and Atg3. Upon completion of the autophagosome, LC3 remains in the autophagosomal lumen (thus serving as an autophagosomal marker), whereas the Atg12–Atg5–Atg16 complex dissociates from the outer autophagosomal membrane. The outer membrane of the autophagosome eventually fuses with the lysosome for degradation of the autophagosomal contents and membrane (10).

Autophagy was originally identified as a mechanism for maintaining homeostasis through the degradation of long-lived proteins and recycling of intracellular organelles (11). However, autophagy is now recognized as playing multiple roles in various biological processes. For example, dysregulation of autophagy has been linked to many diseases, including cancer. Recent studies have revealed that PRRs activate autophagy to enhance immune responses against pathogens and that PRR-induced signaling pathways are regulated by autophagy to prevent excessive inflammation. In this review, we focus on the interactive role of PRRs and autophagy in controlling innate immune responses.

## TLRs and Autophagy

**Figure 1:**
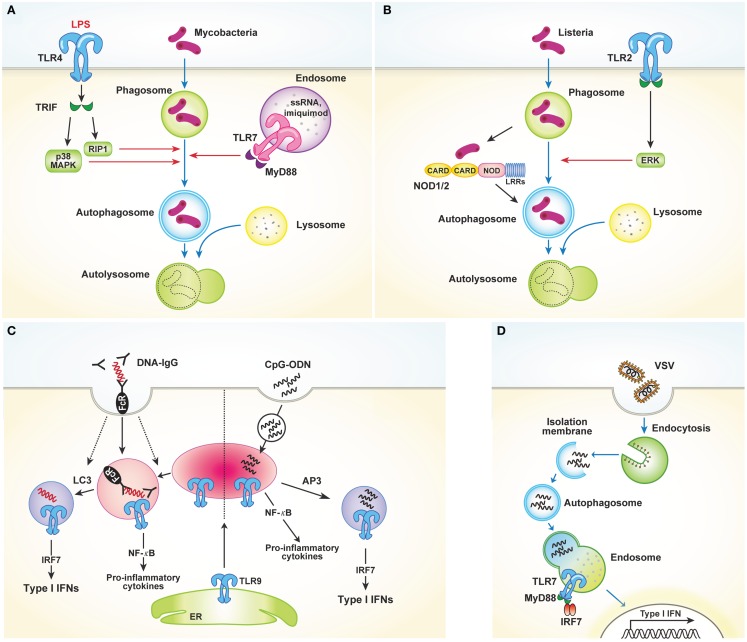
**TLRs and autophagy. (A)** TLR4 stimulated with LPS induces autophagosome formation via TRIF–p38 mitogen-activated protein kinase (MAPK) signaling axis. Similarly, TLR7 activation by ssRNA and imiquimod also promotes autophagy induction. These processes facilitate fusion of the autophagosomes with the lysosomes, which in turn finally result in the killing of intracellular mycobacteria. **(B)** TLR2 and NOD1/2 mediate autophagy induction in response to *Listeria monocytogenes*. Autophagy induction in this process requires the ERK pathway. **(C)** LAP mediates the production of type I IFNs induced by TLR9 activation in response to DNA-ICs. Large DNA-ICs engulfed by phagocytosis are internalized using FcγR, which recruits LC3 and TLR9–UNC93B to the phagosomes. Unlike NF-κB-dependent inflammatory cytokine production, LC3 is required for the trafficking of TLR9 into a specialized IRF7 signaling compartment for type I IFNs secretion. In response to the endocytosed CpG-ODN, however, AP3 is required for delivery to the IRF7 signaling compartment. **(D)** Autophagy facilitates the viral sensing by delivery of cytosolic viral PAMPs to lysosomes, enabling endosomal TLRs to sense of virus and subsequently activating type I IFNs production.

Toll-like receptors, which bind to conserved microbial molecular structures and initiate downstream signaling pathways, are the most thoroughly characterized type of PRR (1). Xu et al. (12) were the first to report that TLR4 stimulation activates autophagy to enhance elimination of phagocytosed mycobacteria. The authors found that stimulation of TLR4 with lipopolysaccharide (LPS) induces autophagosome formation in primary human macrophages and RAW 264.7 murine macrophages. This pathway is mediated by the TRIF–p38 axis rather than MyD88 (Figure 1A). In their study, Xu et al. provide an evidence of close relationship between autophagy and TLR-mediated innate immunity. In addition to LPS-induced autophagy, ligands of TLR3 and TLR7 also activate autophagy. Two different ligands of TLR7, single-stranded RNA (ssRNA) and imiquimod, induce autophagosome formation, characterized by LC3 puncta formation in murine macrophages [Figure 1A; Ref. (13)]. This process occurs via MyD88 and ultimately results in the killing of intracellular mycobacteria, even though mycobacteria are normally not associated with TLR7 signaling.

Recently, several studies reported that TLR2 stimulation by various pathogens induces autophagy (14, 15). In response to *Listeria monocytogenes*, macrophages deficient in the TLR2 and NOD/receptor-interacting protein 2 (RIP2) pathways show defective autophagy induction and fail to colocalize bacteria within autophagosomes [Figure 1B; Ref. (14)]. Autophagy induction in this process was found to be dependent on the extracellular signal-regulated kinase (ERK) pathway. Another study showed that *Staphylococcus aureus*-mediated stimulation of TLR2 in RAW 264.7 mouse macrophages induces phagocytosis and autophagy. In particular, knockdown of *TLR2* was shown to attenuate *S. aureus*-induced phosphorylation of macrophage c-Jun N-terminal kinase (JNK) but not phosphorylation of p38 or ERK (15). Collectively, these data indicate that TLR2 stimulated by invading microbes could mediate autophagy induction and promote the clearance of pathogens, despite the different pathways involved. Shi and Kehrl (16) revealed that various TLR agonists, including TLR1, TLR3, TLR5, TLR6, and TLR7, trigger autophagy induction through MyD88 and TRIF, which interacts with beclin-1. Beclin-1 is critical for the initiation of autophagosome formation. Interaction of beclin-1 with TLR-signaling pathway adaptor molecules partially inhibits the binding of beclin-1 to B cell lymphoma 2 (Bcl-2).

In addition to its role in autophagy induction, TLR-signaling is also utilized by Atg proteins to mediate phagosome maturation. Phagocytosis of the fungal cell wall component zymosan promotes the rapid recruitment of LC3 to phagosomes and facilitates their fusion with lysosomes (17). In RAW 264.7 cells, phagocytosis of Pam_3_CSK_4_-coated latex beads involves recruitment of LC3 to the phagosomes. This process is dependent on TLR2 but not MyD88 and requires both Atg5 and Atg7. However, LC3 translocation to phagosomal membranes is not associated with double-membrane structures, which is a unique feature of autophagosomes. Collectively, these results demonstrate a novel way in which the autophagic machinery is utilized for phagocytosis after TLR activation. Another recent study characterized the role of non-canonical autophagy in type I IFN secretion in response to DNA-immune complexes (DNA-ICs) (18). Upon stimulation of TLR9, which responds to double-stranded DNA (dsDNA) and facilitates the production of proinflammatory cytokines and type I IFNs, IFN-α is produced by the convergence of the phagocytic and autophagic pathways, a process termed LC3-associated phagocytosis (LAP). LAP occurs in response to DNA-ICs but not soluble ligands. In addition, LAP requires FcγR engagement, which controls TLR9 and LC3 recruitment (Figure 1C). The study of Henault et al. revealed the function of non-conventional autophagy in regulating type I IFN signaling in phagosomes. Moreover, their results suggest a mechanism for the uncontrolled production of type I IFNs induced by pathogenic DNA-ICs in systemic lupus erythematosus, which may lead to development of new therapeutic targets for treating this disease.

As previously discussed, induction of autophagy through TLR activation directly promotes pathogen clearance to enhance host protection. However, autophagy also enhances antiviral defenses by facilitating delivery of cytosolic viral PAMPs to endosomal TLRs. Viral nucleic acids endocytosed by host cells are recognized by endosomal TLR7 and TLR9. After recognition, signaling through NF-κB and IRF7 induces production of proinflammatory cytokines and type I IFNs, respectively. In response to vesicular stomatitis virus (VSV) infection in plasmacytoid dendritic cells (pDCs), endosomal TLR7 recognizes the replicating virus in the cytosol rather than the viral genome. How these cytosolic replication intermediates gain access to endosomal TLR7 was demonstrated by Lee and colleagues. These authors showed that autophagy facilitates the delivery of cytosolic PAMPs to the lysosomes, activating TLR7 signaling [Figure 1D; Ref. (19)]. Consequently, pDCs lacking Atg5 cannot secrete IFN-α or IL-12p40 following VSV infection. Atg5-deficient mice are also susceptible to systemic VSV infection *in vivo*. Interestingly, in pDCs infected with herpes simplex virus-1 (HSV-1), which is recognized by TLR9, Atg5-deficient cells fail to produce IFN-α, whereas the IL-12 response of these cells is not affected. Thus, the precise mechanisms by which the NF-κB and IFN-α signaling pathways are controlled by autophagy remain to be determined (20).

## NLRs and Autophagy

**Figure 2:**
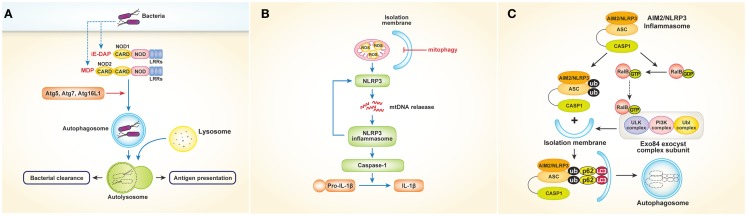
**Interactions between NLRs or inflammasomes and autophagy. (A)** Activation of NOD1 and NOD2 by bacteria induces autophagosome formation, which leads to facilitating bacterial clearance and MHC class II-associated antigen presentation. In this process, autophagy proteins such as Atg5, Atg7, and Atg16L1 are required. **(B)** Autophagy (especially mitophagy) regulates NLRP3 inflammasomes- induced inflammatory responses by quality control of mitochondrial integrity. Blocking mitophagy leads to the accumulation of damaged, ROS-generating mitochondria, which in turn activates NLRP3 inflammasomes. The NLRP3 inflammasome also contributes to the release of mtDNA into the cytosol, enhancing further activation of NLRP3 inflammasomes in a feed-forward circuitry. This process finally activates caspase-1 and results in the excessive production of IL-1β and IL-18. **(C)** Autophagy induced by inflammatory signals targets ubiquitinated inflammasomes, thereby limiting IL-1β production by destruction of inflammasomes. Induction of AIM2 or NLRP3 inflammasomes triggers the activation of RalB to bind to Exo84, which serves as a platform for the formation of isolation membranes. Autophagy engulfs ubiquitinated, assembled inflammasomes through autophagic adaptors such as p62, in turn limiting inflammasome activity.

NOD-like receptors, which recognize bacterial cell wall components such as peptidoglycan in the cytosol, also play an important role in autophagy. Studies have shown that NOD1 and NOD2 activate autophagy in response to bacterial invasion (21, 22). In mouse embryonic fibroblasts (MEFs), NOD2 recruits Atg16L1 to the plasma membrane at the site of bacterial entry, in turn facilitating bacterial trafficking to the autophagosomes and fusion of the autophagosomes with the lysosomes to promote bacterial clearance and antigen presentation via MHCII [Figure 2A; Ref. (22)]. Another study using human DCs showed that stimulation of NOD2 with muramyl dipeptide induces autophagosome formation and consequently enhances MHCII-associated antigen presentation. In this process, autophagic proteins such as Atg5, Atg7, Atg16L1, and receptor-interacting serine–threonine kinase 2 are required [Figure 2A; Ref. (21)]. The intracellular bacterial sensors NOD1 and NOD2 link the autophagic machinery via Atg16L1, thereby enhancing both bacterial clearance and protective immunity. However, the role of Atg16L1 in NOD-derived inflammation remains unclear. A recent study demonstrated that Atg16L1 suppresses NOD-induced inflammatory responses in an autophagy-independent manner (23). Atg16L1 blocks the activation of RIP2 by reducing the level of RIP2 polyubiquitination and diminishing the incorporation of RIP2 into NOD signaling complexes. This process appears to be specific to Atg16L1, given that knockdown of *Atg5* or *Atg9a* does not affect the NOD response. In addition, autophagy-incompetent truncated forms of Atg16L1 retain the capacity to regulate NOD-driven cytokine responses. Interestingly, NOD2 mutations and single-nucleotide polymorphisms in *Atg16L1* are well-known features of Crohn’s disease. Collectively, the above-mentioned studies suggest that a functional relationship exists between NOD2 and Atg16L1 in Crohn’s disease.

Inflammasomes are protein complexes in which NLRs serve as sensory proteins that promote innate immunity by enabling the maturation of pro-IL-1β and pro-IL-18 through activation of pro-caspase-1. Many studies have described regulation of inflammasomes by autophagy and vice versa. Suppression of inflammasomes by autophagy was first reported in 2008 by Saitoh et al. (24), who showed that Atg16L1-deficiency results in increased production of IL-1β and IL-18 following LPS stimulation. Atg16L1 is an essential component of the autophagosome, forming a complex with Atg5–Atg12 conjugates, resulting in LC3–PE conjugation. Thus, Atg16L1-deficient macrophages impaired in autophagosome formation induce TRIF-dependent activation of caspase-1, leading to excessive production of IL-1β in response to LPS. Considering that *Atg16L1* is an important gene in the development of Crohn’s disease, endotoxin-induced inflammasome activation in Atg16L1-deficiency could be involved in the occurrence of Crohn’s disease. Although the above-mentioned data suggest that inflammatory responses are regulated by autophagy, the mechanism by which autophagy regulates cytokine secretion is not clear. Two fascinating studies have provided evidence indicating that mitochondria play a critical role controlling innate immunity mediated by NLRP3 inflammasomes (25, 26). Zhou and colleagues demonstrated that blocking autophagy, especially mitophagy (mitochondrial autophagy), results in the accumulation of damaged, reactive oxygen species (ROS)-generating mitochondria, which in turn activate NLRP3 inflammasomes. Of note, inhibition of mitochondrial activity suppresses both ROS generation and inflammasome activation [Figure 2B; Ref. (26)]. Similarly, Nakahira et al. (25) showed that depletion of the autophagic proteins LC3B and beclin-1 induce excessive secretion of IL-1β and IL-18, which is mediated by accumulation of dysfunctional mitochondria and cytosolic translocation of mitochondrial DNA (mtDNA) following LPS and adenosine triphosphate (ATP) stimulation. The NALP3 inflammasome, which is critical for the activation of caspase-1 in response to LPS and ATP stimulation, contributes to the release of mtDNA into the cytosol [Figure 2B; Ref. (25)]. Together, these studies indicate that regulation of NLRP3-induced inflammatory processes by autophagy is dependent on mitochondrial integrity.

Autophagy also limits the inflammatory responses resulting from inflammasome activation in another way. A recent study showed that autophagy induced by inflammatory signals targets ubiquitinated inflammasomes, thereby limiting IL-1β production through inflammasome destruction (27). Induction of absent in melanoma 2 (AIM2) or NLRP3 inflammasomes triggers nucleotide exchange on RalB and autophagosome assembly through Exo84, which serves as a platform for the formation of isolation membranes (28). During autophagy, ubiquitinated assembled inflammasomes are engulfed through autophagic adaptors such as p62, which contain both ubiquitin-associated domains and LC3-interacting regions that recognize ubiquitinated molecules and assist their entry into the autophagy pathway (Figure 2C). Thus, activation of inflammasomes induces autophagy, which in turn limits inflammasome activity via autophagic engulfment in order to maintain homeostasis as it pertains to inflammation.

Conversely, NLRs also negatively regulate autophagy. NLRC4, NLRP3, NLRP4, and NLRP10 interact with beclin-1, and NLRP4 in particular has a strong affinity for beclin-1. Following invasion by bacteria such as group A streptococci (GAS), NLRP4 recruits GAS-containing phagosomes and transiently dissociates from beclin-1, enabling the initiation of beclin-1-mediated autophagy. Moreover, NLRP4 physically interacts with the class C vacuolar protein-sorting complex, resulting in inhibition of autophagosome and endosome maturation (29). Taken together, the available data indicate that homeostasis is maintained through reciprocal regulation of NLR activation and autophagy.

## Other Cytosolic Sensors and Autophagy

**Figure 3:**
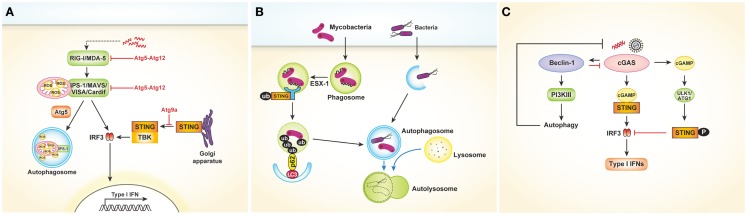
**Cytosolic nucleic acids sensors and autophagy. (A)** Autophagy negatively regulates type I IFNs production after viral infection. The Atg5–Atg12 conjugates directly interact with the CARD domains of RIG-I and IPS-1, inhibiting subsequent RLR signaling pathway and type I IFNs production. In another way, autophagy regulates RLR signaling by acting as a scavenger of dysfunctional mitochondria as well as mitochondria-associated IPS-1. Following dsDNA stimulation, STING is translocated from the ER to the Golgi apparatus and assembled with TBK1, which phosphorylates the transcription factor IRF3. During this process, Atg9a colocalizes with STING in the Golgi apparatus and controls the assembly of STING. **(B)** During mycobacterial clearance, ubiquitin-mediated autophagy targeting *M. tuberculosis* is shown to be activated by the SITNG-dependent cytosolic sensing pathway. Mycobacterial extracellular DNA, which is exposed to the host through ESX-1-mediated permeabilization of the phagosomal membrane, is recognized by the STING-dependent cytosolic pathway. The ubiquitinated bacterial DNA, which binds to the autophagosome-associated protein LC3 via adaptor protein p62 and NDP52, is targeted to the selective autophagy pathway. **(C)** Cytosolic DNA-sensing cGAS produces cGAMP, which binds to and activates the adaptor protein STING, thus leading to the production of type I IFNs. Direct interaction between cGAS and Beclin-1 suppresses cGAMP synthesis. Moreover, this interaction activates PI3K III-induced autophagy, enhancing the autophagy-mediated degradation of pathogen DNA. cGAMP generated by cGAS initially activate STING-dependent type I IFN responses. However, they subsequently trigger negative-feedback control of STING activity through phosphorylation of STING by serine/threonine ULK1 (ATG1).

Viral recognition in most cell types is mediated by cytosolic sensors such as RIG-I and MDA-5. RIG-I and MDA-5, both of which are RLRs, signal through IPS-1 to activate the transcription factors IRF3 and NF-κB, leading to cytokine production. Several studies have revealed that the RLR signaling pathway might be controlled by autophagy (30, 31). In Atg5- or Atg7-deficient MEFs, which lack Atg5–Atg12 conjugates, type I IFNs are overproduced following VSV infection. In contrast, overexpression of Atg5 or Atg12 results in suppression of IFN signaling. The Atg5–Atg12 conjugates directly interact with the CARD domains of RIG-I and IPS-1, inhibiting subsequent RLR signaling [Figure 3A; Ref. (30)]. These data indicate that autophagy-related proteins act as negative regulators of RLR-mediated antiviral responses. Similarly, Tal and colleagues revealed that Atg5-deficient cells overproduce IFNs through enhanced RLR signaling in response to VSV infection (31). However, the authors explained that dysfunctional mitochondria and mitochondria-associated IPS-1 that accumulate in the absence of autophagy enhance RLR signaling. Data suggest that ROS associated with dysfunctional mitochondria are the primary inducers of these responses, as increased mitochondrial ROS production following treatment with rotenone, which is independent of autophagy, also results in amplification of RLR signaling [Figure 3A; Ref. (31)]. Consequently, autophagy contributes to homeostatic regulation of antiviral responses through control of RLR signaling pathways.

The cytosolic DNA sensor stimulator of IFN genes (STING) is also associated with autophagy. In a study to determine the mechanism of mycobacterial clearance, ubiquitin-mediated autophagy targeting *M. tuberculosis* was shown to be activated by the STING-dependent cytosolic sensing pathway (32). In case of wild-type *M. tuberculosis*, which expresses the virulence factor extra-embryonic spermatogenic homeobox 1 (ESX-1) secretion system, mycobacterial DNA may be exposed to the host through ESX-1-mediated permeabilization of the phagosomal membrane. The released DNA may in turn be recognized by the STING-dependent cytosolic pathway. The bacteria are consequently surrounded by ubiquitin chains, and the ubiquitin and LC3-binding autophagic adaptors p62 and nuclear dot protein 52 recruit autophagy components that target the bacilli to the selective autophagy pathway (Figure 3B). Other studies involving dsDNA viruses such as HSV-1 or human cytomegalovirus revealed that STING plays a role in autophagy induction (33, 34).

Conversely, autophagy may also negatively regulate STING-dependent IFN responses. After dsDNA stimulation, Atg9a colocalizes with STING in the Golgi apparatus, where it controls the assembly of STING (35). The loss of Atg9a, but not that of Atg7, promotes the translocation of STING from the Golgi apparatus and its assembly with TBK1, thus inducing aberrant activation of type I IFN responses (Figure 3A). Collectively, these findings demonstrate the reciprocal regulation of autophagy and STING-dependent cytosolic pathways.

Recently, cyclic guanosine monophosphate–adenosine monophosphate (GMP–AMP) synthase (cGAS) was shown to be a cytosolic DNA sensor that activates the type I IFN pathway (36). Cytosolic DNA-sensing cGAS produces cyclic GMP–AMP (cGAMP), which binds to and activates the adaptor protein STING, thus leading to the production of type I IFNs. A very recent study showed that direct interaction between cGAS and beclin-1 suppresses cGAMP synthesis, leading to dampened type I IFN responses following dsDNA stimulation or HSV-1 infection. Moreover, this interaction activates PI3K III-induced autophagy through release of Rubicon, a negative regulator of autophagy, thus enhancing the autophagy-mediated degradation of pathogen DNA to prevent excessive immune stimulation [Figure 3C; Ref. (37)]. Similarly, cyclic dinucleotides contribute to the negative regulation of the STING pathway by activating UNC-51-like kinase (ULK1/Atg1). Cyclic dinucleotides generated by cGAS initially activate STING-dependent type I IFN responses; however, they subsequently trigger negative-feedback control of STING activity through phosphorylation of STING by serine/threonine ULK1/Atg1 [Figure 3C; Ref. (38)]. Taken together, these data suggest that autophagy controls the excessive and persistent immune responses mediated by cytosolic DNA-sensing pathways.

## Conclusion

In this review, we describe the close interaction between PRRs and autophagy in various immunologic conditions. PRRs are not only involved in autophagy induction but also in the promotion of phagosomal maturation mediated by Atg proteins when pathogenic bacteria invade host cells. In addition, autophagy facilitates the delivery of viral PAMPs and TLR9 trafficking for type I IFN production. Autophagy regulates PRR-induced inflammation in various ways to prevent excessive inflammatory responses, and conversely, PRR signaling also controls autophagy. Collectively, the available data indicate that targeting autophagy would allow us to enhance pathogen clearance or suppress PRR-mediated inflammatory conditions, such as those associated with autoimmune diseases. Therefore, a more detailed analysis of how we could control autophagy is recommended.

## Conflict of Interest Statement

The authors declare that the research was conducted in the absence of any commercial or financial relationships that could be construed as a potential conflict of interest.
